# The Epitope of Monoclonal Antibodies Blocking Erythrocyte Invasion by *Plasmodium falciparum* Map to The Dimerization and Receptor Glycan Binding Sites of EBA-175

**DOI:** 10.1371/journal.pone.0056326

**Published:** 2013-02-15

**Authors:** Xavier Ambroggio, Lubin Jiang, Joan Aebig, Harold Obiakor, Jan Lukszo, David L. Narum

**Affiliations:** 1 Bioinformatics and Computational Biosciences Branch, Office of Cyber Infrastructure and Computational Biology, National Institute of Allergy and Infectious Diseases, National Institutes of Health, Bethesda, Maryland, United States of America; 2 Institut Pasteur of Shanghai, Chinese Academy of Sciences, Shanghai, China; 3 Laboratory of Malaria Immunology and Vaccinology, National Institute of Allergy and Infectious Diseases, National Institutes of Health, Rockville, Maryland, United States of America; 4 Research Technologies Branch, National Institute of Allergy and Infectious Diseases, National Institutes of Health, Rockville, Maryland, United States of America; Johns Hopkins Bloomberg School of Public Health, United States of America

## Abstract

The malaria parasite, *Plasmodium falciparum*, and related parasites use a variety of proteins with Duffy-Binding Like (DBL) domains to bind glycoproteins on the surface of host cells. Among these proteins, the 175 kDa erythrocyte binding antigen, EBA-175, specifically binds to glycophorin A on the surface of human erythrocytes during the process of merozoite invasion. The domain responsible for glycophorin A binding was identified as region II (RII) which contains two DBL domains, F1 and F2. The crystal structure of this region revealed a dimer that is presumed to represent the glycophorin A binding conformation as sialic acid binding sites and large cavities are observed at the dimer interface. The dimer interface is largely composed of two loops from within each monomer, identified as the F1 and F2 β-fingers that contact depressions in the opposing monomers in a similar manner. Previous studies have identified a panel of five monoclonal antibodies (mAbs) termed R215 to R218 and R256 that bind to RII and inhibit invasion of erythrocytes to varying extents. In this study, we predict the F2 β-finger region as the conformational epitope for mAbs, R215, R217, and R256, and confirm binding for the most effective blocking mAb R217 and R215 to a synthetic peptide mimic of the F2 β-finger. Localization of the epitope to the dimerization and glycan binding sites of EBA-175 RII and site-directed mutagenesis within the predicted epitope are consistent with R215 and R217 blocking erythrocyte invasion by *Plasmodium falciparum* by preventing formation of the EBA-175– glycophorin A complex.

## Introduction

Malaria as a clinical disease is due to the complex cyclical process of merozoite invasion of red blood cells (RBCs). The merozoite proteins or ligands involved in this process are localized primarily in three subcellular locations: the merozoite surface, rhoptries and micronemes. Merozoite proteins function during invasion in a coordinated manner while subject to an active immune response and phenotypic variation of human erythrocytes.

The 175 kDa erythrocyte binding antigen (EBA-175) was one of the earliest micronemal proteins identified that bound human erythrocytes and whose function could be blocked by antibodies [1]. Within EBA-175, a region identified as RII contains two Duffy Binding Like (DBL) domains called F1 and F2 that are responsible for binding glycophorin A in a sialic acid dependent manner [2]–[4]. A recombinant EBA-175 RII protein [5] was crystallized in the form of a dimer [6], indicating that EBA-175 dimerization may be biologically important for receptor binding and RBC invasion. The role of human antibodies against proteins containing DBL domains such as EBA-175 [7], [8], EBA-140 (BAEBL/EBP2) [9]–[11], EBA-181/JESEBL [12], and *P. vivax* DBP [13]–[16] in clinical immunity is unclear. Human antibodies to EBA-175 RII are associated with clinical protection [17], but *in vitro* studies designed to assess blocking activity show that, individually, the antibodies incompletely interfere with erythrocyte invasion [7]. The incomplete blocking activity could be the result of low antibody titers [7] or due to the presence of redundant biological mechanisms for erythrocyte invasion [16], [18]–[20].

In order to investigate the function of EBA-175 RII, a panel of five monoclonal antibodies (mAbs) specific to EBA-175 RII was generated, three of which (R215, R217, R256) were found to be specific and compete for the F2 domain of RII in the native, disulfide-bonded form. All three of these mAbs potently blocked binding of EBA-175 to erythrocytes and merozoite invasion of erythrocytes, with R217 and R256 demonstrating the greatest biological effect [21]. One of the mAbs, R216, was found to recognize F2 in reduced form and failed to effectively block binding of native EBA-175 to erythrocytes. The remaining mAb R218 was specific for the F1 domain, and inhibited parasite growth with considerably less effectiveness than the F2 specific antibodies [21].

In the present study, the epitopes of the F2 specific mAbs were predicted through structural and bioinformatic analysis of peptides generated through phage display experiments and characterized experimentally for mAb binding. We further investigated the functional importance of the predicted epitope region through site-directed mutagenesis. The results suggest that erythrocyte binding inhibition by these mAbs is due to interference in the formation of the EBA-175 dimer – glycophorin A receptor complex.

## Materials and Methods

### Antibodies

The characterization of the EBA-175 RII (3D7) specific mouse mAbs R215, R216, R217, and R256 has been reported [21]. Briefly, R215, R217 and R256 recognize the F2 domain of RII by immune-blotting in the native, disulfide-bonded form, while R216 recognizes the reduced form. Only mAbs R215, R217 and R256 inhibit *P. falciparum* growth, *in vitro*. Purified IgG from each clone was prepared as previously described [21].

### Screening of the Phage Display Library

The Ph.D.-C7C™, a disulfide constrained random 7-mer phage display library, and Ph.D.-12™, a linear random 12-mer phage display library were screened by panning on mAb as recommended by the manufacturer (New England BioLabs, Beverly, MA, USA). Following a third or fourth round of amplification, individual phage clones were isolated and the peptide sequences they displayed were deduced following DNA sequencing.

### Computational Epitope Mapping Using Peptides from Phage Display Libraries

PepSurf [22] and Mapitope [23] were used to map the peptides found in the phage display libraries for mAb R215, R217, and R256 to the crystal structure of EBA-175 RII (PDB: 1ZRO) [6]. The PepSurf and Mapitope results for the R215, R217, and R256 mAbs were cross-referenced with each other and those positions identified for all three mAbs were defined as putative epitope residues. These residues were used to guide a visual analysis of the crystal structure to select the peptide fragment for experimental characterization. Solvent accessible surface area calculations for the predicted epitopes were performed with AREAIMOL of CCP4 [24].

### Peptide Synthesis and Preparation

The F2 β-finger (F2βf) peptide (VWECKKPYKLSTKDVCVPP), corresponding to amino acids 473 through 491 from the EBA-175 3D7 sequence (NCBI accession number: CAD51055) and amino acids 329 to 347 of the recombinant EBA-175 (rEBA-175) crystal structure, was synthesized by a solid-phase method using Fmoc chemistry on an automated peptide synthesizer (Model 433A, Applied Biosystems, Life Technologies Corporation, Carlsbad, CA, USA). After the trifluoroacetic acid cleavage step, synthetic peptide was purified to homogeneity by reversed-phase (RP) high-performance liquid chromatography (HPLC). The purified, linear peptide was oxidized to a cyclic form (cyclic F2βf) containing an intramolecular disulfide bond, between cysteines 4 and 16 of the peptide, utilizing 2,2′-dithiodipyridine [25] in standard buffer solution. The mass of the cyclic F2βf peptide was confirmed by Matrix-Assisted Laser Desorption Ionization – mass spectrometry.

A linearized version of F2βf (linear F2βf) was created by reduction and alkylation. F2βf was reduced using a 1 M stock solution of dithiothreitol (DTT) that was added to the peptide for a final concentration of 5 mM DTT. The mixture was incubated at room temperature for 1 hour. A 0.8 M stock solution of iodoacetamide was added to the reduced peptide for a final concentration of 0.2 M iodoacetamide. The mixture was incubated in the dark at room temperature for 1 hour. The reduced and alkylated peptide was characterized by reversed-phase ultra-performance liquid chromatography and was stored frozen at −70°C until use.

### RII and F2βf Enzyme-linked Immunosorbent Assays (ELISA)

Details of the enzyme-linked immunosorbent assay (ELISA) have been described [26]. Briefly, the EBA-175 RII protein [5], the linear, or cyclic F2βf peptides were coated onto Immulon 4HBX flat-bottom microtiter plates (DynexTechnologies, Chantilly, VA, USA) at 10 µg/ml in phosphate-buffered saline (PBS), pH 7.4. Following blocking in buffered skim milk, purified mAbs (IgG), diluted in 0.1% bovine serum albumin/Tris-buffered saline, were added. Two mAbs, R216 and R256, were tested as hybridoma culture supernatants and diluted in the same buffer beginning with a 1∶2 dilution. For detection, a solution of anti-mouse IgG (H+L) antibodies conjugated to alkaline phosphatase (KPL, Inc., Gaithersburg, MD, USA) diluted 1∶1600 was used. Substrate (Phosphatase substrate, Sigma) developed in the plates for either 20 min (for EBA-175 RII-coating) or 60 min (for F2βf-coating). End-point dilutions were considered positive for those wells with an absorbance at 405 nm 2-fold greater than the control wells. The absorbance at 405 nm was read in a SpectraMax 190 Microplate Reader (Molecular Devices, LLC, Sunnyvale, CA, USA).

### Surface Plasmon Resonance (SPR) Binding Studies

Biacore 3000 instrument (GE Healthcare, Piscataway, NJ) was used for SPR data acquisition. Reactive N-hydroxysuccinimide esters were introduced on CM5 sensor chip (GE Healthcare) through modification of the carboxymethylated dextran hydrogel by an 8 min injection of a mixed solution of 0.4 M 1-ethyl-3-(3-dimethylaminopropyl) carbodiimide (EDC) and 0.1 M N-hydroxysuccinimide (NHS) (GE Healthcare). Amine coupling chemistry was used to covalently immobilize 20 µg/ml of EBA-175 RII diluted in 10 mM sodium acetate, pH 5.5 on test flow cell 2 by injection over the succinimide esters activated surface for 10 min. A 7 min injection of 1 M ethanolamine, pH 8.5 was used to block any remaining reactive esters on the surface of the CM5 sensor chip. Reference flow cell 1 was similarly prepared in the absence of protein. The flow rate for the coupling reaction was 10 µl/min and the detection temperature was 25°C. Immobilization levels achieved were 12730 RU for EBA-175 RII and 152 RU on the reference flow cell 1. Both flow cells were equilibrated with HBS-EP [0.01 M HEPES, pH7.4, 0.15 M NaCl, 3 mM EDTA, 0.005% Surfactant P20] (GE Healthcare) before protein analysis. Monoclonal antibodies R215, R217 and R218 against EBA-175 RII were each diluted to 33.3 nM in HBS-EP and injected over flow cells 1 and 2 at 30 µl/min. Then 33.3 nM of each mAb was incubated in 90.0 µM, 45.0 µM, 22.5 µM and 11.3 µM dilutions of cyclic F2βf peptide in HBS-EP for 30 min and injected over flow cells 1 and 2 at 30 µl/min. The amount of mAb bound to the immobilized EBA-175 RII was obtained by subtracting the response (RU) on the reference flow cell 1 from that on the test flow cell 2. Injection of 20 µl of 10 mM Glycine-HCl, pH 2.5 was sufficient to regenerate both flow cells to levels similar to the starting baseline after each binding experiment. Binding interactions were determined using BIAevaluation software (version 4.1.1) (GE Healthcare).

### Erythrocyte Binding Assays

The pRE4 plasmid [27] containing the gene sequence for EBA-175 RII Camp [4] was modified at amino acid position 566 (422 in rEBA-175 RII) from arginine to alanine or lysine by Retrogen, Inc., (San Diego, CA, USA). The sequences of the modified genes encoding EBA-175 RII were DNA sequenced to verify their integrity.

Normal erythrocytes (O^+^ type) obtained from the Interstate Blood Bank (Memphis, TN) were processed to remove serum and leucocytes and stored at 4°C as described previously [28]. Neuraminidase treatment of the erythrocytes was done as described previously [29]. Erythrocyte binding assays were performed with CHO-K1 cells (American Type Culture Collection, Manassas, VA, USA) according to the previous report [29]. CHO-K1 cells were grown to 90–95% confluency in 6-well plates and transfected with 4 µg of each plasmid DNA using lipofectamine 2000, according to the manufacturer’s instructions (Invitrogen, Carlsbad, CA, USA). After 20–24 hours, 2×10^5^ transfected cells were incubated with 10 µl of erythrocytes in 800 µl of RPMI medium 1640 with 10% fetal calf serum, 4 mM glutamine, penicillin (100 units/ml), streptomycin (100 µg/ml) and 10 mM HEPES (Invitrogen, Carlsbad, CA, USA) pH 7.55 at 37°C for 4 hours. To remove unbound erythrocytes, media was removed and each well was washed gently several times with the media described above. Transfected CHO-K1 cells with rosettes of at least 5 bound erythrocytes in each well were counted under a Leica DM IRE2 inverted microscope (Leica Microsystems Wetzlar GmbH, Wetzlar, Germany). pT8 vector containing wild-type EBA-175 RII or PfEMP1 DBL2 domain was used as control [29].

## Results

### Phage Display

A cyclic (disulfide-bond constrained) random 7-mer peptide phage-display library was screened to identify peptides that interacted with the RII specific mAbs R215, R217 and R256. A linear random peptide display library was used for screening RII specific mAb R216. The deduced amino acid sequences derived from the phage clones, selected by panning against the mAbs, are shown in Table 1 and the corresponding nucleotide sequences are shown in Table S1.

**Table 1:** Deduced amino acid sequences of clones enriched for each EBA-175 RII specific mAb.

Constrained 7-mers			Linear 12-mer
R215	R217	R256	R216
KWWLMNS	PISKLHL	NMVPMSR	HSNSSWISRQTY
KWWLMPP	PISKLHL	NMVPLWR	GNSLHNYPRGPT
KWWLMPP	PISKLHL	TMVPMWR	TRPFEPIQALFK
KWWLMPP	PQSKLHL	WSINPRW	VKLHPSSLVSLN
KWWIMPP	KTPALKH	WSINPRW	SVVTPQTLSSGS
KWWIMPP	IQHRGPA	WSINPRW	SYFDVTPFRSRA
WWQSKLR	TIPLPWH	WSINPRW	GALHVPNMYHVV
PWHKTRY	TLSFPHR	WSINPRW	HLYPKTEEALLR
NPFGPFY	PTTFLNA	NTMTQMY	MNTYQLVGDQPP
QTTGMLA	NTHLLKG	ESRTEYR	QSHLRFWYDHQT

### Epitopes of F2 Specific mAbs Computationally Map to F2 β-finger Region

**Figure 1:**
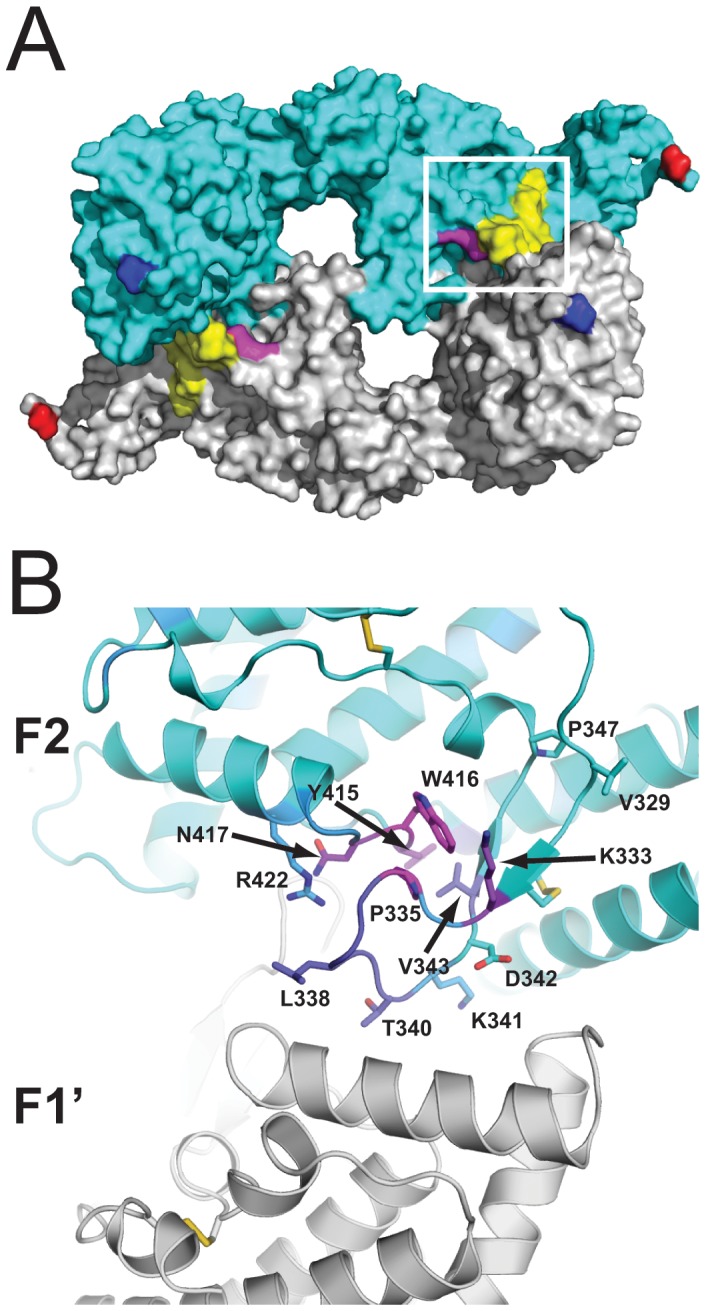
Mapping of epitope predictions to the rEBA-175 RII crystal structure. (A) rEBA-175 RII crystal structure as a dimer of two RII molecules (cyan and grey), the region corresponding to the F2βf peptide is highlighted in yellow and R422 is highlighted in magenta. The amino- and carboxy- terminal residues are colored in blue and red respectively. The white box represents the region highlighted in panel B. (B) The F2 domain of one monomer in the rEBA-175 RII crystal structure is shown as a ribbon diagram and residues are colored by the number of times they were predicted by either PepSurf and Mapitope to be part of the epitope for mAbs R215, R217, or R256 from never (cyan) to most often (magenta; see SOM for raw data). Residues discussed in the text are labeled according to rEBA-175 RII numbering (EBA-175 3D7 sequence number –144) and shown in stick representation.

The F2 β-finger region encompasses the F2 β-finger, a β-hairpin module bounded by a disulfide between C476 and C488 (C332 and C344 of rEBA-175 RII), and a structurally adjacent loop between helices R and S, from approximately I554 to R566 (I410 to R422 of rEBA-175 RII) [6]. Three residue positions, P479, Y559, and W560 (P335, Y415, and W416 of rEBA-175 RII), were predicted by PepSurf and/or Mapitope to belong to the epitopes of mAbs R215, R217, and R256 (Figure 1, Text S1, and Figure S1). Residues K477 and N561 (K333 and N417 of rEBA-175 RII) were predicted in multiple clusters to belong to the epitope of R256. The former residue, K477, was also predicted to belong to the epitope of R215 and the latter residue, N561, was also predicted to belong to the epitope of R217. These two sets of positions (477, 479 and 559–561) are spatially adjacent in the crystal structure of EBA-175 RII (Figure 1) with a total contact area of 102 Å^2^ between them.

### R215 and R217 mAbs bind a F2 β-finger Derived Peptide

**Figure 2:**
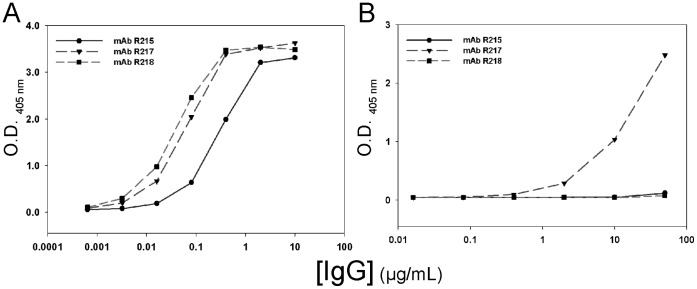
Binding characteristics of EBA-175 RII specific mAbs R215, R217 and R218 to recombinant EBA-175 RII (A) or disulfide-constrained F2βf peptide (B) by ELISA.

**Figure 3:**
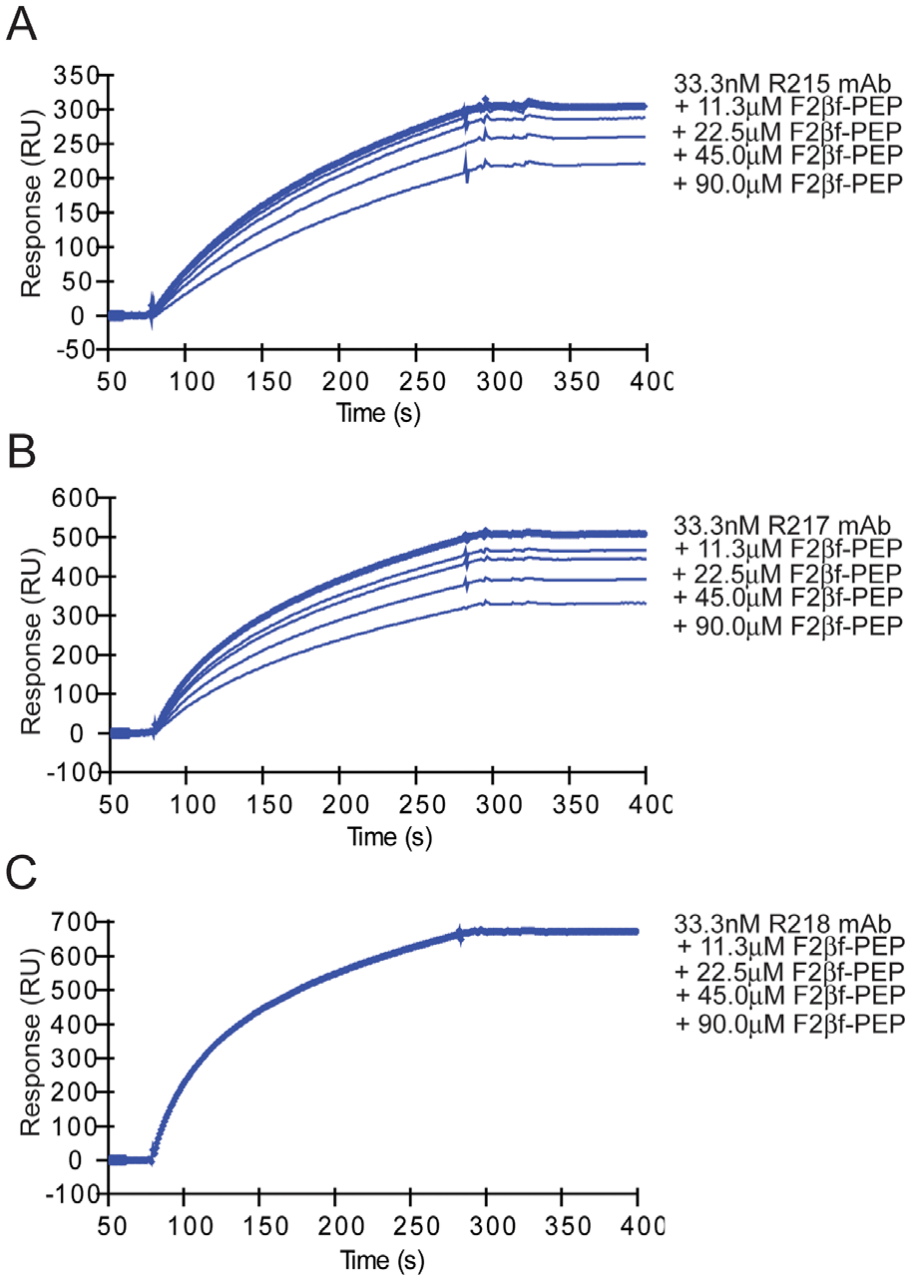
F2βf peptide competes with mAbs R215 (A) and R217 (B) but not R218 (C) for binding to recombinant EBA-175 RII by Surface Plasmon Resonance studies. Monoclonal antibody sensograms show binding to immobilized EBA-175 RII with the concentrations of mAb and cyclic F2βf peptide as indicated. The concentrations of cyclic F2βf peptide were added to a fixed concentration of mAb.

Results for peptide binding assays are summarized in Table 2. ELISAs were used to evaluate the binding of purified IgG of mAbs R215 and R217 or hybridoma supernatants of mAbs R216 and R256 to EBA-175 RII, cyclic, and linear F2βf peptides. Purified mAbs R215 and R217 showed reactions to EBA-175 RII at concentrations greater than 0.016 µg/mL and 0.003 µg/mL, respectively (Figure 2A). MAbs R216 and R256 specific IgG in culture supernatants also reacted well to EBA-175 RII (data not shown). The four mAbs were tested for binding to cyclic F2βf, and the results for R215 and R217 are shown in Figure 2B. Monoclonal antibody R217 showed reactions at concentrations greater than 0.4 µg/ml, while R215 was weakly positive at 50 µg/mL. Monoclonal antibodies R216 and R256 did not exhibit binding to the cyclic or linear form of F2βf (data not shown), however the failure of R256 binding to the cyclic F2βf could be due its lower concentration in culture supernatant. In addition, R217 did not bind linear F2βf. MAbs R215, R217 and R218 bound specifically to EBA-175 RII by SPR analysis (Figure 3). Cyclic F2βf peptide competed with mAbs R215 (Figure 3A) and R217 (Figure 3B) for binding to EBA-175 RII in a concentration dependent manner but not with mAb R218 (Figure 3C).

**Table 2:** Summary of binding properties of EBA-175 RII specific mAbs.

mAb	RIIdomain [21]	Epitope	Cyclic F2βf binding**	
			ELISA	SPR
R215	F2^c^	Conformational	±	+
R216*	F2	Linear	−	ND
R217	F2^c^	Conformational	+	+
R218	F1	Conformational	−	−
R256	F2^c^	Conformational	−	ND

SPR: surface plasmon resonance. ND: not determined.

* Does not inhibit parasite growth *in vitro* [21].

** None of the mAbs demonstrated binding to linear F2βf.

c These mAbs were shown to compete against each other for binding by competition ELISA.

### Erythrocyte Binding Assays

Arginine was reported to be important in the binding specificity of a member of the DBL family, BAEBL [29]. To investigate if the arginine in the predicted epitope region, R566 (R422 of rEBA-175 RII), plays a role in binding of EBA-175 RII to erythrocytes, we mutated this amino acid to alanine and expressed the recombinant protein on the surface of CHO-K1 cells for erythrocyte binding assays. The resulting EBA-175 RII mutant (R566A) bound poorly to normal erythrocytes compared to wild type EBA-175 RII (Table 3). As expected, no binding was observed in the negative control DBL-2 (Table 3). Additionally, we evaluated a conservative arginine to lysine substitution at this position (R566K). Like wild type EBA-175 RII, the new EBA-175 mutant (R566K) strongly bound normal erythrocytes. The transfection efficiency evaluated by using a monoclonal antibody recognizing a C-terminal epitope in the pT8 vector [29] or pRE4 vector [27] was 60–70% in all constructs in CHO-K1 cells (data not shown).

**Table 3:** Effect of arginine mutation on EBA-175 RII binding to human erythrocytes.

	Binding of EBA-175 RII mutants to human erythrocytes^a^		
	Normal erythrocytes^b^		Nm erythrocytes^c^
	Exp. 1	Exp. 2	
DBL-2	4	1	13
EBA-175 RII	234	410	9
R566A*	52	112	28
R566K*	248	328	20

a the rosette number was an average counted from two biological repeats in each experiment.

b erythrocyte-binding assay was performed with normal erythrocytes in two independent experiments.

c erythrocyte-binding assay was performed with neuraminidase-treated erythrocytes Nm, neuraminidase.

* Residue 566 of EBA-175 corresponds to residue 422 of rEBA-175 RII.

It has been shown that EBA-175 RII binds erythrocyte receptor glycophorin A requiring sialic acids on glycophorin A. To investigate if mutations of R566 alter binding specificity of EBA-175 to erythrocytes, we used neuraminidase-treated erythrocytes to further test the mutated EBA-175 RII. Neuraminidase treatment removes sialic acids on the surface of erythrocytes, resulting in failure of EBA-175 binding to enzyme-treated erythrocytes [28]. Our data showed that the R566A mutant bound poorly to all erythrocytes tested, whereas the R566K mutant and wild type EBA-175 RII bound poorly only to neuraminidase-treated erythrocytes (Table 3).

## Discussion

Phage display coupled to computational mapping predicts the β-finger region of the EBA-175 RII F2 domain as the conformational epitope for mAbs R215, R217, and R256. The β-finger region encompasses the F2 β-finger, a β-hairpin module bounded by a disulfide between C476 and C488 (C332 and C344 of rEBA-175 RII), and a structurally adjacent loop between helices R and S, from approximately I554 to R566 (I410 to R422 of rEBA-175 RII) [6]. The crystal structure and experimental characterization of EBA-175 RII demonstrated a clear role for the F2 β-finger and surrounding F2 region in both dimerization and glycan binding [6]. The mAbs R215, R217, and R256 were previously found to inhibit parasite invasion of erythrocytes, with mAb R217 displaying the highest level of potency for inhibition [21].

The F2 β-finger forms a significant portion of the dimer interface of rEBA-175 RII and four residues within the F2 β-finger T484–V487 (T340–V343 of rEBA-175 RII) were previously implicated in binding to glycans 5 and 6 of glycophorin A [6]. For mAbs R215 and R217, mapping of the F2 β-finger portion of the predicted epitope was experimentally verified by SPR competition assays. Binding of mAbs R215 and R217 to EBA-175 RII was diminished in a concentration-dependent manner by pre-incubation with a synthetic, disulfide-constrained peptide encompassing the F2 β-finger (cyclic F2βf). Binding of mAb R217 to cyclic F2βf was also observed by ELISA, while mAb R215 was weak. The antibody concentration needed to elicit a signal between R217 and cyclic F2βf by ELISA was two orders of magnitude larger than the antibody concentration needed to elicit a signal between R217 and EBA-175 RII. The comparative weakness of cyclic F2βf in eliciting a signal by ELISA for R217, and the associated weak signal for R215 and the absence of signal for R256, may result from differences in coating, and or the cyclic F2βf peptide representing a partially structured epitope in coated form, or an incomplete epitope with respect to R256.

Our computational mapping predicted that a second loop spatially adjacent to the F2 β-finger, I554–R566 (I410–R422 of rEBA-175 RII), may also form a significant part of the epitope for R215, R217, and R256. For this study, we were unable to synthesize a peptide encompassing this loop to test in particular for mAb R256 binding; however, we investigated the functional significance of this loop, as it may be impaired either directly through mAb binding or sterically through mAb binding of the adjacent F2 β-finger. Residues N561 and R566 (N417 and R422 of rEBA-175 RII) in this loop were previously predicted to bind glycans 1 and 2 of glycophorin A and an R566E substitution was found to abolish binding of the mutant EBA-175 RII to normal human erythrocytes [6]. Here, we extend those findings to show that binding of EBA-175 RII to erythrocytes is dependent on the presence of a positive charge at residue 566. We found that an R566K substitution was sufficient to restore wild-type binding levels from the absence of binding observed by Tolia *et al.* with R566E [6] or the reduction of binding observed here with R566A. R566 is widely conserved in RII among *P. falciparum* strains [30]. Among the known sialic acid receptors, both neuraminidase and Siglec receptors rely on the positive charge from arginine(s) to bind negatively charged sialic acids via charge neutralization [31], [32]. Our data suggest the similar mechanism utilized by EBA-175 for ligand recognition of glycophorin A, in which the positive charge of R566 in EBA-175 RII plays a critical role. Shielding of the positive charge at R566 by an antibody, either directly by charge neutralization through residues of the antibody or indirectly by making the charge inaccessible through steric occlusion, would likely mimic the effect of these mutations, resulting in interference of binding of EBA-175 RII to erythrocytes.

Given the localization of the epitopes of R215 and R217 to the dimerization and glycan binding sites of EBA-175 RII, inhibition of erythrocyte invasion by these mAbs is likely mediated by prevention of the EBA-175– glycophorin A complex, however, the precise mechanism will require further investigation. EBA-175 RII was observed to be a monomer at low concentrations and a dimer at high concentrations [6] and the dimeric form is believed to be the state in which RII interacts with glycophorin A. It is unclear however, if the RII dimer assembles around glycophorin A or if glycophorin A binds to a preassembled dimer. Given these possibilities, the simplest mechanism of action for the observed mAb-mediated binding inhibition is that the mAbs bind the F2 β-finger region of RII in the monomeric state, thereby preventing both dimerization and subsequent glycophorin A binding, or glycan-mediated glycophorin A binding directly through steric occlusion. Recently, a stretch of 69 amino acids of the related erythrocyte binding ligand 1 (EBL-1) protein was identified as the binding site for glycophorin B [33]. The second loop of the predicted epitope on EBA-175 aligns within this region of EBL-1 (data not shown). Two mutations (D554A, R485A, rEBA-140 numbering) in residues of EBA-140, within the general region homologous to the mapped epitope of EBA-175, were found to abolish binding to its cellular receptor, glycophorin C [34]. In contrast to EBA-175 and *P. vivax* DBP, EBA-140 does not appear to have a dimeric state and binds to its receptor as a monomer [34]. Like EBA-175, EBA-140 elicits antibodies capable of inhibiting erythrocyte invasion [12]. EBL-1, EBA-140, and EBA-175 all appear to use the region mapped here as an epitope on EBA-175 for binding to cellular receptors, despite low sequence conservation in this region.

In conclusion, the β-finger region of RII/F2 is predicted to be the epitope recognized by the EBA-175 specific mAbs R215, R217, and R256 and experimental binding to a synthetic peptide epitope mimic of the F2 β-finger was demonstrated for mAbs R215 and R217. The F2 β-finger region encompasses a key charged amino acid, R566, which appears largely responsible for RBC binding to CHO-K1 cells expressing RII. Mapping of the epitope to the F2 β-finger region will facilitate further advancements in mechanistic studies evaluating the interactions between EBA-175 and glycophorin A binding upon host cell invasion by the malaria parasite, and most importantly, the development of assays such as a competitive ELISA to assess the possible function of natural [17] and vaccine [35] induced human EBA-175 RII specific antibodies. The mechanism by which EBA-175 RII antibodies are protective *in vivo* is still unknown. The epitope mapping presented here, will enable the development of additional tools for elucidating the mechanisms behind clinical protection.

## Supporting Information

Figure S1 **Pepsurf** [**1**] **and Mapitope** [**2**] **residue counts.** In the chart, bar height represents the number of times a residue (given on the X-axis) was predicted to be part of the epitope by Pepsurf or Mapitope based on the phage display peptides (see data below) generated for R256 (green), R217 (maroon), or R215 (blue). Residues are numbered according to the numbering in the crystal structure of rEBA-175 RII [3].
(PNG)

Table S1 Phage display nucleotide sequences and deduced amino acid sequences for EBA-175 RII mAb panel presented in Table 1.
(DOCX)

Text S1 **Raw Pepsurf** [**1**] **and Mapitope** [**2**] **data using the peptides presented in** **Table 1** **and the crystal structure of rEBA-175 RII** [**3**] **with residues numbered accordingly.**
(DOCX)

References S1 (DOCX)
