# Supplementary figures and images for: The Epitope of Monoclonal Antibodies Blocking Erythrocyte Invasion by Plasmodium falciparum Map to The Dimerization and Receptor Glycan Binding Sites of EBA-175
Source: PLoS One. 2013 Feb 15;8(2):e56326. doi: 10.1371/journal.pone.0056326 (PMC3574135; doi:10.1371/journal.pone.0056326)

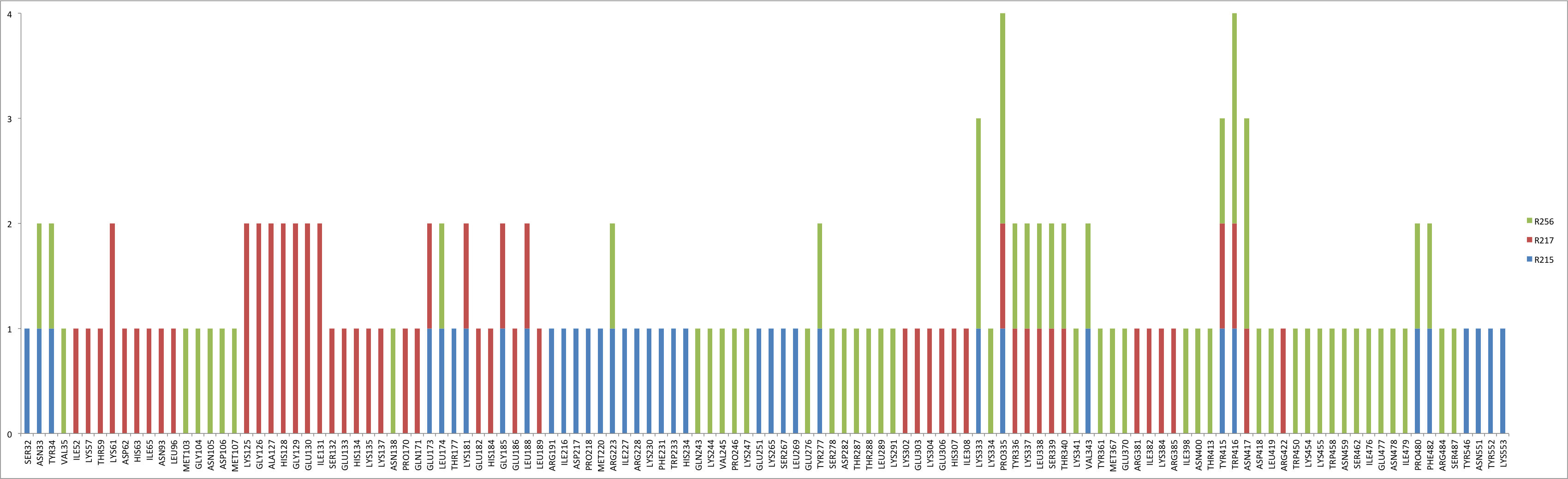

Supplement: Figure S1 — Pepsurf [1] and Mapitope [2] residue counts. In the chart, bar height represents the number of times a residue (given on the X-axis) was predicted to be part of the epitope by Pepsurf or Mapitope based on the phage display peptides (see data below) generated for R256 (green), R217 (maroon), or R215 (blue). Residues are numbered according to the numbering in the crystal structure of rEBA-175 RII [3]. (PNG) [file pone.0056326.s001.png]
