# Supplementary material for: The Epitope of Monoclonal Antibodies Blocking Erythrocyte Invasion by Plasmodium falciparum Map to The Dimerization and Receptor Glycan Binding Sites of EBA-175
Source: PLoS One. 2013 Feb 15;8(2):e56326. doi: 10.1371/journal.pone.0056326 (PMC3574135; doi:10.1371/journal.pone.0056326)
Supplement: Table S1 — Phage display nucleotide sequences and deduced amino acid sequences for EBA-175 RII mAb panel presented in Table 1. (DOCX) [file pone.0056326.s002.docx]

**Table S1.** Phage display nucleotide sequences and deduced amino acid sequences for EBA-175 RII mAb panel presented in Table 1.

| **CLONE** | **DNA sequence of phage insert**  **5’ TO 3’** | **Deduced amino acid sequence** |
| --- | --- | --- |
| **R215** | | |
| 1 | AAGTGGTGGCTTATGAATTCC | KWWLMNS |
| 2 | AAGTGGTGGTTGATGCCTCCT | KWWLMPP |
| 3 | AAGTGGTGGTTGATGCCTCCT | KWWLMPP |
| 4 | AAGTGGTGGTTGATGCCTCCT | KWWLMPP |
| 5 | AAGTGGTGGATTATGCCTCCT | KWWIMPP |
| 6 | AAGTGGTGGATTATGCCTCCT | KWWIMPP |
| 7 | TGGTGGCAGTCGAAGCTTCGT | WWQSKLR |
| 8 | CCTTGGCATAAGACGCGGTAT | PWHKTRY |
| 9 | AATCCTTTTGGGCCGTTTTAT | NPFGPFY |
| 10 | CAGACTACGGGGATGCTGGCG | QTTGMLA |
| **R217** | | |
| 1 | CCGCAGAGTAAGCTTCATTTG | PISKLHL |
| 2 | CCTATTTCGAAGCTTCATCTT | PISKLHL |
| 3 | CCTATTTCGAAGCTTCATCTT | PISKLHL |
| 4 | CCTATTTCGAAGCTTCATCTT | PQSKLHL |
| 5 | AAGACGCCGGCTCTTAAGCAT | KTPALKH |
| 6 | ATTCAGCATCGGGGTCCGGCT | IQHRGPA |
| 7 | ACGATTCCTCTGCCTTGGCAT | TIPLPWH |
| 8 | ACGCTTTCGTTTCCTCATCGG | TLSFPHR |
| 9 | cctacgacgtttctgaatgct | PTTFLNA |
| 10 | AATACTCATCTTCTGAAGGGT | NTHLLKG |
| **R256** | | |
| 1 | AATATGGTTCCGATGTCGCGT | NMVPMSR |
| 2 | AATATGGTTCCGCTGTGGAGG | NMVPLWR |
| 3 | ACTATGGTTCCTATGTGGAGG | TMVPMWR |
| 4 | TGGTCGATTAATCCGCGTTGG | WSINPRW |
| 5 | TGGTCGATTAATCCGCGTTGG | WSINPRW |
| 6 | TGGTCGATTAATCCGCGTTGG | WSINPRW |
| 7 | TGGTCGATTAATCCGCGTTGG | WSINPRW |
| 8 | TGGTCGATTAATCCGCGTTGG | WSINPRW |
| 9 | AATACGATGACTTAGATGTAT | NTMTQMY |
| 10 | GAGTCTCGGACGGAGTATCGG | ESRTEYR |
| **R216** | | |
| 1 | CATTCGAATTCTTCTTGGATTTCGCGTCAGACTTAT | HSNSSWISRQTY |
| 2 | GGTAATAGTCTGCATAATTATCCTCGGGGGCCGACG | GNSLHNYPRGPT |
| 3 | ACTCGGCCTTTTGAGCCTATTCAGGCTCTGTTTAAG | TRPFEPIQALFK |
| 4 | GTTAAGCTTCATCCGAGTTCTCTGGTTTCGCTGAAT | VKLHPSSLVSLN |
| 5 | TCGGTTGTTACGCCTCAGACTTTGTCTAGTGGGTCT | SVVTPQTLSSGS |
| 6 | TCGTATTTTGATGTGACGCCGTTTCGGTCTCGTGCG | SYFDVTPFRSRA |
| 7 | GGGGCGCTGCATGTTCCGAATATGTATCATGTTGTT | GALHVPNMYHVV |
| 8 | CATCTTTATCCTAAGACTGAGGAGGCGCTTCTGCGT | HLYPKTEEALLR |
| 9 | ATGAATACTTATCAGCTGGTTGGTGATCAGCCGCCG | MNTYQLVGDQPP |
| 10 | CAGTCGCATCTGCGTTTTTGGTATGATCATCAGACT | QSHLRFWYDHQT |
