# Supplementary material for: The Epitope of Monoclonal Antibodies Blocking Erythrocyte Invasion by Plasmodium falciparum Map to The Dimerization and Receptor Glycan Binding Sites of EBA-175
Source: PLoS One. 2013 Feb 15;8(2):e56326. doi: 10.1371/journal.pone.0056326 (PMC3574135; doi:10.1371/journal.pone.0056326)
Supplement: Text S1 — Raw Pepsurf [1] and Mapitope [2] data using the peptides presented in Table 1 and the crystal structure of rEBA-175 RII [3] with residues numbered accordingly. (DOCX) [file pone.0056326.s003.docx]

**Text S1.** Raw Pepsurf [[1](#_ENREF_1)] and Mapitope [[2](#_ENREF_2)] data using the peptides presented in Table 1 and the crystal structure of rEBA-175 RII [[3](#_ENREF_3)] with residues numbered accordingly.

R215 mapitope.cluster:

ILE216:A

ASP217:A

PRO218:A

MET220:A

R215 pepsurf.cluster:

Best cluster

Score: 42.744

Residues number: 5

LYS333:A

PRO335:A

VAL343:A

TYR415:A

TRP416:A

---------------------------------------------

Peptides participating in this cluster:

ID Sequence

2:R215_1 KWWLMPP

3:R215_2 KWWLMPP

4:R215_3 KWWLMPP

5:R215_4 KWWIMPP

6:R215_5 KWWIMPP

R215 pepsurf.cluster2:

Cluster rank: 2

Score: 36.075

Residues number: 22

SER32:A

ASN33:A

TYR34:A

GLU173:A

LEU174:A

THR177:A

LYS181:A

GLY185:A

LEU188:A

ARG191:A

ARG223:A

ILE227:A

ARG228:A

LYS230:A

PHE231:A

TRP233:A

HIS234:A

GLU251:A

LYS265:A

SER267:A

LEU269:A

TYR277:A

---------------------------------------------

Peptides participating in this cluster:

ID Sequence

1:R215_0 KWWLMNS

7:R215_6 WWQSKLR

8:R215_7 PWHKTRY

10:R215_9 QTTGMLA

R215 pepsurf.cluster3:

Cluster rank: 3

Score: 9.6979

Residues number: 6

PRO480:A

PHE482:A

TYR546:A

ASN551:A

TYR552:A

LYS553:A

---------------------------------------------

Peptides participating in this cluster:

ID Sequence

9:R215_8 NPFGPFY

R217 combined.cluster:

Cluster rank: 1

Residues number: 7

intersects cluster number 3 of PepSurf with cluster number 1 of Mapitope

GLU130:A

ILE131:A

LYS125:A

HIS128:A

GLY126:A

GLY129:A

ALA127:A

R217 combined.cluster2:

Cluster rank: 2

Residues number: 1

intersects cluster number 3 of PepSurf with cluster number 3 of Mapitope

LYS61:A

R217 mapitope.cluster:

Best cluster

Residues number: 6

LYS125:A

GLY126:A

ALA127:A

HIS128:A

GLY129:A

GLU130:A

ILE131:A

SER132:A

GLU133:A

HIS134:A

LYS135:A

LYS137:A

R217 mapitope.cluster2:

Cluster rank: 2

Residues number: 6

LYS302:A

GLU303:A

LYS304:A

GLU306:A

HIS307:A

ILE308:A

ARG381:A

ILE382:A

LYS384:A

ARG385:A

R217 mapitope.cluster3:

Cluster rank: 3

Residues number: 4

ILE52:A

LYS61:A

ASP62:A

HIS63:A

ILE65:A

R217 pepsurf.cluster:

Best cluster

Score: 48.946

Residues number: 12

ASN93:A

LEU96:A

PRO170:A

GLN171:A

GLU173:A

LYS181:A

GLU182:A

HIS184:A

GLY185:A

GLU186:A

LEU188:A

LEU189:A

---------------------------------------------

Peptides participating in this cluster:

ID Sequence

1:R217_0 PISKLHL

2:R217_1 PISKLHL

3:R217_2 PISKLHL

4:R217_3 PQSKLHL

10:R217_9 NTHLLKG

R217 pepsurf.cluster2:

Cluster rank: 2

Score: 20.063

Residues number: 10

PRO335:A

TYR336:A

LYS337:A

LEU338:A

SER339:A

THR340:A

TYR415:A

TRP416:A

ASN417:A

ARG422:A

---------------------------------------------

Peptides participating in this cluster:

ID Sequence

7:R217_6 TIPLPWH

8:R217_7 TLSFPHR

R217 pepsurf.cluster3:

Cluster rank: 3

Score: 16.369

Residues number: 10

LYS57:A

THR59:A

LYS61:A

LYS125:A

GLY126:A

ALA127:A

HIS128:A

GLY129:A

GLU130:A

ILE131:A

---------------------------------------------

Peptides participating in this cluster:

ID Sequence

5:R217_4 KTPALKH

6:R217_5 IQHRGPA

R256 combined.cluster:

Cluster rank: 1

Residues number: 4

intersects cluster number 3 of PepSurf with cluster number 3 of Mapitope

ASN417:A

LYS333:A

TRP416:A

PRO335:A

R256 mapitope.cluster:

Best cluster

Residues number: 12

ASN33:A

TYR34:A

VAL35:A

MET103:A

GLY104:A

ASN105:A

ASP106:A

MET107:A

ASN138:A

LEU174:A

GLN243:A

LYS244:A

VAL245:A

PRO246:A

LYS247:A

R256 mapitope.cluster2:

Cluster rank: 2

Residues number: 9

ILE398:A

ASN400:A

ILE476:A

GLU477:A

ASN478:A

ILE479:A

PRO480:A

PHE482:A

ARG484:A

SER487:A

R256 mapitope.cluster3:

Cluster rank: 3

Residues number: 8

LYS333:A

LYS334:A

PRO335:A

TYR336:A

LYS337:A

LEU338:A

SER339:A

THR340:A

LYS341:A

TRP416:A

ASN417:A

ASP418:A

LEU419:A

R256 pepsurf.cluster:

Best cluster

Score: 45.364

Residues number: 6

TRP450:A

LYS454:A

LYS455:A

TRP458:A

ASN459:A

SER462:A

---------------------------------------------

Peptides participating in this cluster:

ID Sequence

4:R256_3 WSINPRW

5:R256_4 WSINPRW

6:R256_5 WSINPRW

7:R256_6 WSINPRW

8:R256_7 WSINPRW

R256 pepsurf.cluster2:

Cluster rank: 2

Score: 19.459

Residues number: 12

ARG223:A

GLU276:A

TYR277:A

SER278:A

ASP282:A

THR287:A

THR288:A

LEU289:A

LYS291:A

TYR361:A

MET367:A

GLU370:A

---------------------------------------------

Peptides participating in this cluster:

ID Sequence

9:R256_8 NTMTQMY

10:R256_9 ESRTEYR

R256 pepsurf.cluster3:

Cluster rank: 3

Score: 18.654

Residues number: 7

LYS333:A

PRO335:A

VAL343:A

THR413:A

TYR415:A

TRP416:A

ASN417:A

---------------------------------------------

Peptides participating in this cluster:

ID Sequence

2:R256_1 NMVPLWR

3:R256_2 TMVPMWR
