# Supplementary material for: The Epitope of Monoclonal Antibodies Blocking Erythrocyte Invasion by Plasmodium falciparum Map to The Dimerization and Receptor Glycan Binding Sites of EBA-175
Source: PLoS One. 2013 Feb 15;8(2):e56326. doi: 10.1371/journal.pone.0056326 (PMC3574135; doi:10.1371/journal.pone.0056326)
Supplement: References S1 — (DOCX) [file pone.0056326.s004.docx]

**References S1**

[1] Mayrose I, Shlomi T, Rubinstein ND, Gershoni JM, Ruppin E, Sharan R, et al. Epitope mapping using combinatorial phage-display libraries: a graph-based algorithm. Nucleic Acids Res. 2007;35:69-78.

[2] Bublil EM, Freund NT, Mayrose I, Penn O, Roitburd-Berman A, Rubinstein ND, et al. Stepwise prediction of conformational discontinuous B-cell epitopes using the Mapitope algorithm. Proteins. 2007;68:294-304.

[3] Tolia NH, Enemark EJ, Sim BK, Joshua-Tor L. Structural basis for the EBA-175 erythrocyte invasion pathway of the malaria parasite Plasmodium falciparum. Cell. 2005;122:183-93.
